# Outcomes in participants with failure of initial antibacterial therapy for hospital-acquired/ventilator-associated bacterial pneumonia prior to enrollment in the randomized, controlled phase 3 ASPECT-NP trial of ceftolozane/tazobactam versus meropenem

**DOI:** 10.1186/s13054-022-04192-w

**Published:** 2022-12-01

**Authors:** Marin H. Kollef, Jean-François Timsit, Ignacio Martin-Loeches, Richard G. Wunderink, Jennifer A. Huntington, Erin H. Jensen, Brian Yu, Christopher J. Bruno

**Affiliations:** 1 Division of Pulmonary and Critical Care Medicine, Washington University School of Medicine, St Louis, MO USA; 2 Intensive Care Medicine Department, Université Paris Diderot and Hôpital Bichat, Paris, France; 3 Department of Intensive Care Medicine, Multidisciplinary Intensive Care Research Organization (MICRO), St James’ Hospital, Dublin, Ireland; 4 Hospital Clinic, Universitat de Barcelona, IDIBAPS, CIBERES, Barcelona, Spain; 5 Pulmonary and Critical Care Division, Northwestern University Feinberg School of Medicine, Chicago, IL USA; 6 Merck & Co., Inc., Rahway, NJ USA

**Keywords:** Nosocomial pneumonia, HABP, VABP, Mechanical ventilation, All-cause mortality, Clinical response, Multivariable analysis, Refractory, Failing prior antibacterial therapy

## Abstract

**Background:**

Ceftolozane/tazobactam, a combination antibacterial agent comprising an anti-pseudomonal cephalosporin and β-lactamase inhibitor, is approved for the treatment of hospital-acquired/ventilator-associated bacterial pneumonia (HABP/VABP) in adults. Participants in the ASPECT-NP trial received ceftolozane/tazobactam (3 g [2 g ceftolozane/1 g tazobactam] every 8 h) or meropenem (1 g every 8 h). Participants failing prior antibacterial therapy for the current HABP/VABP episode at study entry had lower 28-day all-cause mortality (ACM) rates with ceftolozane/tazobactam versus meropenem treatment. Here, we report a post hoc analysis examining this result.

**Methods:**

The phase 3, randomized, controlled, double-blind, multicenter, noninferiority trial compared ceftolozane/tazobactam versus meropenem for treatment of adults with ventilated HABP/VABP; eligibility included those failing prior antibacterial therapy for the current HABP/VABP episode at study entry. The primary and key secondary endpoints were 28-day ACM and clinical response at test of cure (TOC), respectively. Participants who were failing prior therapy were a prospectively defined subgroup; however, subgroup analyses were not designed for noninferiority testing. The 95% CIs for treatment differences were calculated as unstratified Newcombe CIs. Post hoc analyses were performed using multivariable logistic regression analysis to determine the impact of baseline characteristics and treatment on clinical outcomes in the subgroup who were failing prior antibacterial therapy.

**Results:**

In the ASPECT-NP trial, 12.8% of participants (93/726; ceftolozane/tazobactam, *n* = 53; meropenem, *n* = 40) were failing prior antibacterial therapy at study entry. In this subgroup, 28-day ACM was higher in participants who received meropenem versus ceftolozane/tazobactam (18/40 [45.0%] vs 12/53 [22.6%]; percentage difference [95% CI]: 22.4% [3.1 to 40.1]). Rates of clinical response at TOC were 26/53 [49.1%] for ceftolozane/tazobactam versus 15/40 [37.5%] for meropenem (percentage difference [95% CI]: 11.6% [− 8.6 to 30.2]). Multivariable regression analysis determined concomitant vasopressor use and treatment with meropenem were significant factors associated with risk of 28-day ACM. Adjusting for vasopressor use, the risk of dying after treatment with ceftolozane/tazobactam was approximately one-fourth the risk of dying after treatment with meropenem.

**Conclusions:**

This post hoc analysis further supports the previously demonstrated lower ACM rate for ceftolozane/tazobactam versus meropenem among participants who were failing prior therapy, despite the lack of significant differences in clinical cure rates.

*ClinicalTrials.gov registration* NCT02070757. Registered February 25, 2014, clinicaltrials.gov/ct2/show/NCT02070757.

**Supplementary Information:**

The online version contains supplementary material available at 10.1186/s13054-022-04192-w.

## Introduction

Hospital-acquired/ventilator-associated bacterial pneumonia (HABP/VABP) is one of the most common healthcare-acquired infections reported among patients in the intensive care unit [1–5]. Patients with nosocomial pneumonia are often critically ill, with mortality rates ranging from approximately 20–30% [6, 7]. Multidrug resistance has been associated with multifold increases in the risk of hospital-related mortality and is considered to be an important factor in the failure of initially appropriate antibacterial therapy for gram-negative pathogens [8]. Delayed microbiologically appropriate antibacterial therapy and microbiologic failure of antibacterial therapy are associated with increased morbidity and mortality [9, 10]. In addition, co-resistance to first-line β-lactams among carbapenem-resistant pathogens may impact outcomes if not considered during selection of second-line therapies [11]. Analysis of recent isolates from lower respiratory tract (LRT) infections collected in the USA indicated significantly higher incidence of resistant phenotypes among gram-negative isolates from intensive care unit patients compared with non-intensive care unit patients [12–14]. The rise of multidrug resistance among key gram-negative pathogens, such as *Pseudomonas aeruginosa* and Enterobacterales, has been recognized as a global public health issue owing to elevated risk of mortality among critically ill patients, highlighting the need for new treatment options for HABP/VABP [15–17].

Ceftolozane/tazobactam, a combination antibacterial agent comprising the anti-pseudomonal cephalosporin, ceftolozane, and the β-lactamase inhibitor active against extended-spectrum β-lactamases (ESBLs), tazobactam, is broadly active in vitro against multiple pathogens associated with HABP/VABP [18]. Activity in vitro has been demonstrated against common LRT pathogens, as well as multidrug-resistant gram-negative LRT pathogens, including *P. aeruginosa* and ESBL-producing Enterobacterales [12, 19–23]. Ceftolozane/tazobactam was approved for treatment in adults with HABP/VABP based on the pivotal, phase 3 ASPECT-NP trial results (NCT02070757), wherein noninferiority of ceftolozane/tazobactam versus meropenem for the treatment of HABP/VABP in ventilated participants was demonstrated for both 28-day all-cause mortality (ACM) and clinical response [24]. Among participants who were failing prior antibacterial therapy for the current HABP/VABP episode, a lower mortality rate was observed in those who received ceftolozane/tazobactam compared with meropenem (22.6% vs 45.0%) [24]. These findings are of clinical interest because patients who are refractory to ≥ 1 first-line antibacterial therapy for HABP/VABP generally have a higher mortality rate compared with those who respond to their initial therapy because they (1) are generally sicker than those who are not refractory, (2) have greater exposure to the health care system, and (3) are more likely to have infections because of multidrug-resistant pathogens [25].

Here, we explore whether failing prior gram-negative therapy impacted efficacy outcomes in the ASPECT-NP trial and, if so, whether clinical or baseline characteristics were associated with 28-day ACM among participants who received ceftolozane/tazobactam or meropenem with a history of failed prior therapy.

## Materials and methods

### Study design and participants

The design of the ASPECT-NP clinical trial (NCT02070757; protocol MK-7625A-008) has been described previously [24]. Briefly, ASPECT-NP was a prospective, randomized, controlled, double-blind, multicenter, phase 3, noninferiority trial assessing ceftolozane/tazobactam versus meropenem for the treatment of adults (aged ≥ 18 years) with ventilated HABP (vHABP)/VABP. Enrollment included intubated, mechanically ventilated participants diagnosed with VABP or vHABP ≤ 24 h before receiving the first dose of ceftolozane/tazobactam or meropenem. Participants with only gram-positive pathogens present on baseline Gram stain were excluded. Participants who had received > 24 h of systemic or inhaled antibacterial agents active against gram-negative pathogens that cause HABP/VABP in the 72 h before enrollment were also excluded [24]. An exception to this exclusion criterion was participants who were determined to be failing prior antibacterial therapy for their current episode of HABP/VABP (based on investigator interpretation of persistent, worsening, or new signs and/or symptoms of nosocomial pneumonia despite ≥ 48 h of gram-negative antibacterial therapy for HABP/VABP), which is the subgroup that is the focus of this manuscript.

Participants who met the eligibility criteria were randomized (1:1) to receive either ceftolozane/tazobactam at twice the dose (3 g [2 g ceftolozane and 1 g tazobactam] every 8 h) approved for other indications or 1 g meropenem every 8 h for a total duration of 8–14 days [26]; administration of amikacin 15 mg/kg for the first 72 h of study treatment was permitted as adjunctive empiric therapy at hospitals with ≥ 15% of *P. aeruginosa* isolates identified as being meropenem resistant. Until confirmed absence of *Staphylococcus aureus* in baseline LRT cultures, adjunctive linezolid 600 mg intravenously (IV) every 12 h was required for all participants. To balance high-risk participants between treatment arms, randomized participants were stratified by vHABP or VABP diagnosis and by age (≥ 65 or < 65 years). All investigators, study staff, and participants/participant representatives were blinded to treatment during the duration of the study; only pharmacists who prepared masked infusion bags were unblinded and were permitted to adjust dosing of study drug based on renal function in accordance with the approved regimens. Participants were discontinued from study drug if they experienced clinical or microbiologic failure requiring any other nonstudy HABP/VABP treatment. LRT cultures were collected at baseline (≤ 36 h before randomization) and post-baseline during the first week of treatment from participants while intubated, at end of therapy (EOT), and at test of cure (TOC; 7–14 days post-EOT). Pathogen identification and susceptibility were confirmed at a central laboratory. Breakpoints for susceptibility to ceftolozane/tazobactam were ≤ 4 μg/mL for Enterobacterales and ≤ 8 μg/mL for *P. aeruginosa*, *Acinetobacter baumannii*, *Haemophilus influenzae*, and other bacterial pathogens. Current Clinical and Laboratory Standards Institute (CLSI) breakpoints were used to determine meropenem susceptibility [27]. Collection of microbiology data was limited to gram-negative and streptococcal LRT pathogens.

Populations included in the analyses were the intention-to-treat (ITT) population (defined as all randomized participants), the clinically evaluable (CE) population (defined as participants who received study drug, adhered to protocol requirements, and had an evaluable clinical outcome at TOC), microbiologic ITT (mITT) population (defined as participants who received ≥ 1 dose of study treatment and had ≥ 1 gram-negative or streptococcal respiratory pathogen from baseline LRT cultures that was susceptible to ≥ 1 study drug), and the microbiologically evaluable (ME) population (defined as participants who received study drug, adhered to protocol requirements, had ≥ 1 gram-negative or streptococcal respiratory pathogen from baseline LRT cultures [at the appropriate colony-forming unit (CFU)/mL threshold: ≥ 10^5^ CFU/mL for endotracheal aspiration, ≥ 10^4^ CFU/mL for bronchoalveolar lavage/mini-bronchoalveolar lavage, and ≥ 10^3^ CFU/mL for protected specimen brush] from the baseline LRT culture that was susceptible to ≥ 1 study drug, and had an evaluable clinical outcome at TOC) [24].

Both the primary endpoint (28-day ACM) and the key secondary endpoint (clinical response at TOC) were assessed in the ITT population. Additional secondary endpoints were clinical response at TOC (CE population), per-pathogen microbiologic response and per-participant microbiologic response at TOC (mITT and ME populations), and 28-day ACM (mITT population). Safety was assessed in all randomized participants who received ≥ 1 dose of study treatment from first dose of study treatment to the late follow-up visit (28–35 days after EOT) according to treatment received [24].

### Subgroup analyses

Participants with vHABP/VABP who were failing prior antibacterial therapy at study entry comprised a prospectively defined subgroup. Treatment failure was determined by the investigator and defined as signs and/or symptoms of the current vHABP/VABP episode persisting or worsening despite treatment with ≥ 48 h of antibacterial therapy potentially effective against gram-negative pathogens that typically cause vHABP/VABP, or signs and/or symptoms of vHABP/VABP that developed after ≥ 48 h of treatment with antibacterial therapy potentially effective against gram-negative organisms that typically cause vHABP/VABP, given for an infection other than the current vHABP/VABP episode. Participants could not be enrolled if they had received > 24 h of carbapenem therapy within the 7 days prior to first dose of study therapy or had growth of a meropenem- or ceftolozane/tazobactam-resistant pathogen from a respiratory (not including the baseline lower respiratory tract culture) or blood culture within 15 days prior to first dose of study drug. Analyses of endpoints for participants with vHABP/VABP who were failing prior antibacterial therapy were performed prospectively (primary and key secondary efficacy endpoints) and retrospectively (all other analyses). We performed a post hoc analysis of baseline clinical and microbiologic factors, treatment factors, efficacy, and safety between treatment arms in participants who were failing prior antibacterial therapy for vHABP/VABP. There was no stratification within subgroup analyses, which were not designed for noninferiority testing; 95% CIs were calculated as unstratified Newcombe CIs [28]. Missing responses, including indeterminates, were either deemed deaths or clinical or microbiologic failures depending on the analysis (ITT and mITT populations) or were excluded from the analysis (CE and ME populations). Safety outcomes were analyzed descriptively. SAS version 9.3 (SAS Institute, Cary, NC, USA) software was used to perform all statistical analyses.

### Multivariable analysis

A multiple logistic regression analysis was performed based on a previously described model [29]. The analysis aimed to evaluate 2 questions: (1) which clinical/microbiologic factors were predictive of 28-day ACM in the subgroup of participants in this trial who were failing prior antibacterial therapy and (2) whether treatment (ceftolozane/tazobactam vs meropenem) still impacted 28-day ACM after adjusting for predictive clinical/microbiologic factors. For this analysis, 16 clinical and microbiologic factors (continuous variables: age, Acute Physiology and Chronic Health Evaluation [APACHE] II score, Sequential Organ Failure Assessment [SOFA] score, Clinical Pulmonary Infection Score [CPIS], and arterial oxygen partial pressure to fractional inspired oxygen [PaO_2_/FiO_2_]; categorical, dichotomous variables [presence vs absence]: vHABP [yes vs VABP], ≥ 5 days of prior hospitalization, ≥ 5 days of prior mechanical ventilation, baseline bacteremia [any pathogen], all baseline pathogens susceptible to randomized study drug, baseline *P. aeruginosa*, baseline ESBL-positive Enterobacterales, adjunctive gram-negative therapy, concomitant vasopressor use, moderate to severe impairment of creatinine clearance [15 to 50 mL/min], and treatment arm [ceftolozane/tazobactam vs meropenem]) were chosen based on clinical input. These factors were chosen because of their potential to affect treatment outcomes and their availability from the collected data.

To address the potential modeling complexities related to inclusion of factors associated with multicollinearity, the random forest ensemble method was selected to determine the ranked relative importance each factor contributes toward predicting mortality within this population. The random forest ensemble method is a decision-tree learning algorithm capable of delineating relationships between variables that are both complex and nonlinear in nature [30, 31]. To perform the analysis, the package randomForest (CRAN; version 4.6–14) in R (CRAN; version 3.6.6) was used on the ITT analysis population who were failing prior antibacterial therapy during the ASPECT-NP trial. Details of the random forest algorithm modeling parameters have been described previously [29]. The 16 prespecified factors were ranked based on relative influence on 28-day ACM and then entered into a logistic regression model using forward variable selection. The factors were entered into the model in order of influence from most influential on 28-day ACM to least influential. The receiver operating characteristic (ROC) was calculated at each step and was used as the metric for assessing factor influence on model prediction. The Hosmer–Lemeshow test was used to determine goodness-of-fit for the model.

Based on the forward variable selection, the factors identified as most influential were then entered into a backward selection main effects logistic regression model to assess the magnitude on predicting 28-day ACM in participants who were failing prior antibacterial therapy. To remain in the backward selection logistic regression model, terms were required to have a *P* value of < 0.05. The final model yielded estimated odds ratios (ORs) and 95% CIs for the increased/decreased potential of 28-day ACM. A separate sensitivity analysis including all 16 factors as main effects was performed using a traditional multivariable logistic regression. SAS version 9.3 (SAS Institute, Cary, NC) software was used to perform all logistic regression analyses [29].

## Results

### Participants

**Fig. 1:**
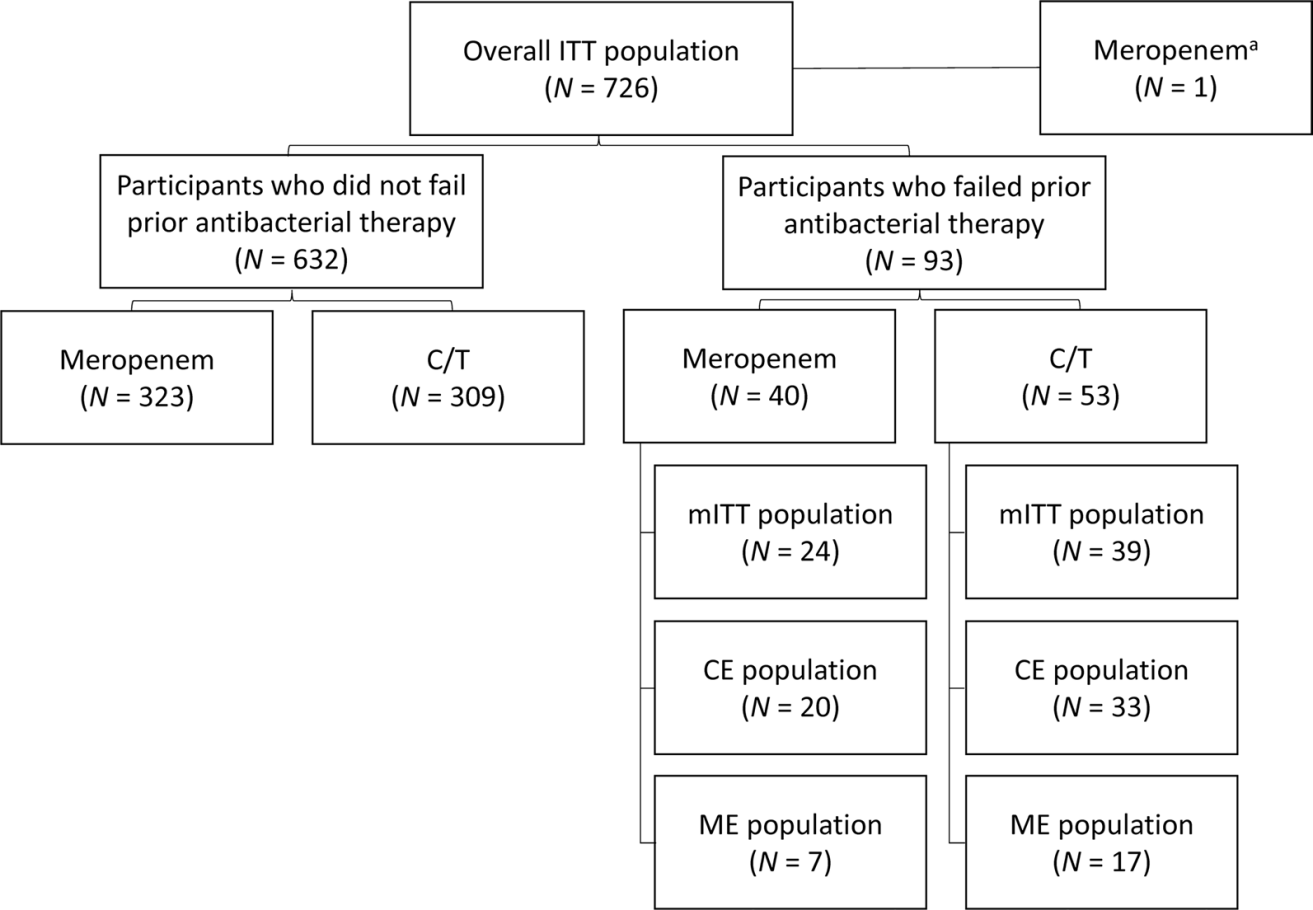
Participant and analysis population flow chart. *CE* Clinically evaluable, *C/T* Ceftolozane/tazobactam, *ITT* Intention-to-treat, *ME* Microbiologically evaluable, *mITT* Microbiologic intention-to-treat, *N* Number of participants in specific analysis population

In the ASPECT-NP trial, 93/726 (12.8%) participants were failing current antibacterial therapy for their ongoing vHABP/VABP episode at randomization; 53/362 (14.6%) ceftolozane/tazobactam-treated and 40/364 (11.0%) meropenem-treated (Fig. 1). The most common systemic antibacterial agents or antibacterial classes these participants received in the 72 h prior to enrollment were similar between the ceftolozane/tazobactam and meropenem treatment arms: piperacillin/tazobactam (30.2% ceftolozane/tazobactam vs 42.5% meropenem), fluoroquinolones (28.3% vs 30.0%), and third-/fourth-generation cephalosporins (24.5% vs 40.0%) (Table 1). When compared with participants who were not failing prior antibacterial therapy, those who were failing prior therapy were more likely to have received mechanical ventilation for ≥ 5 days prior to randomization (58.1% vs 48.1%) and to be diagnosed with vHABP (39.8% vs 26.7%) (Table 2 and Additional file 1: Table S1). Characteristics at baseline within the failing prior therapy subgroup were generally comparable and well balanced between treatment arms; however, a trend toward higher disease severity scores among participants in the ceftolozane/tazobactam arm was observed (Table 2). Compared with those randomized to meropenem at baseline within this subgroup, participants receiving ceftolozane/tazobactam were more likely to have APACHE II scores ≥ 20 (43.4% vs 25.0%), have SOFA scores > 7 (32.1% vs 15.0%), and have PaO_2_/FiO_2_ values ≤ 240 mm Hg (77.4% vs 57.5%).

**Table 1:** Antibacterial treatments received within 72 h prior to starting study treatment in ASPECT-NP participants^a^ who were failing prior antibacterial therapy

Antibacterial treatment, *n* (%)	C/T (*N* = 53)	MEM (*N* = 40)
Piperacillin/tazobactam	16 (30.2)	17 (42.5)
Fluoroquinolones	15 (28.3)	12 (30.0)
Third/fourth-generation cephalosporins	13 (24.5)	16 (40.0)
Cefepime	4 (7.5)	3 (7.5)
Cefotaxime	0	4 (10.0)
Ceftriaxone	9 (17.0)	6 (15.0)
Amoxicillin/clavulanate	6 (11.3)	2 (5.0)
Ampicillin/sulbactam	5 (9.4)	1 (2.5)
Aminoglycosides	7 (13.2)	4 (10.0)
Amikacin	5 (9.4)	4 (10.0)
Macrolides	3 (5.7)	1 (2.5)
Carbapenems	2 (3.8)	3 (7.5)
Cefoperazone/sulbactam	4 (7.5)	3 (7.5)

Includes all systemic antibacterial treatments with potential gram-negative activity administered within 72 h prior to starting study treatment; participants may have received multiple treatments sequentially and/or concomitantly

*C/T* Ceftolozane/tazobactam, *MEM* Meropenem, *vHABP* Ventilated hospital-acquired bacterial pneumonia, *VABP* Ventilator-associated bacterial pneumonia

^a^In ≥ 3 participants

**Table 2:** Baseline characteristics in ASPECT-NP participants who were failing prior antibacterial therapy

	C/T (*N* = 53)	MEM (*N* = 40)	Total (*N* = 93)
Primary diagnosis, *n* (%)			
VABP	33 (62.3)	23 (57.5)	56 (60.2)
vHABP	20 (37.7)	17 (42.5)	37 (39.8)
Sex, *n* (%)			
Male	43 (81.1)	24 (60.0)	67 (72.0)
Female	10 (18.9)	16 (40.0)	26 (28.0)
Age, years			
< 65, *n* (%)	26 (49.1)	20 (50.0)	46 (49.5)
≥ 65, *n* (%)	27 (50.9)	20 (50.0)	47 (50.5)
Mean (SD)	60.2 (18.7)	61.3 (15.6)	60.7 (17.4)
Median (range)	65.0 (21, 88)	63.5 (29, 92)	65.0 (21, 92)
Weight (kg)			
Median (range)	80.0 (34.0, 119.5)	70.5 (45.0, 225.0)	76.0 (34.0, 225.0)
Body-mass index (kg/m^2^)			
Median (range)	26.8 (15.1, 43.3)	25.8 (16.2, 67.2)	26.0 (15.1, 67.2)
Creatinine clearance, mL/min, *n* (%)			
≥ 150 (augmented renal clearance)	9 (17.0)	6 (15.0)	15 (16.1)
≥ 80	35 (66.0)	26 (65.0)	61 (65.6)
< 80 to > 50	11 (20.8)	9 (22.5)	20 (21.5)
≤ 50 to ≥ 30	5 (9.4)	3 (7.5)	8 (8.6)
< 30 to ≥ 15	2 (3.8)	2 (5.0)	4 (4.3)
< 15 (end-stage renal disease)	0	0	0
APACHE II score			
≤ 14, *n* (%)	13 (24.5)	13 (32.5)	26 (28.0)
15–19, *n* (%)	17 (32.1)	17 (42.5)	34 (36.6)
≥ 20, *n* (%)	23 (43.4)	10 (25.0)	33 (35.5)
Mean (SD)	18.1 (5.2)	17.0 (5.2)	17.6 (5.2)
Median (range)	18.0 (7, 32)	16.0 (4, 29)	17.0 (4, 32)
SOFA score, *n* (%)			
≤ 7	36 (67.9)	34 (85.0)	70 (75.3)
> 7	17 (32.1)	6 (15.0)	23 (24.7)
Adjunctive gram-negative therapy,^a^ *n* (%)			
Yes	13 (24.5)	12 (30.0)	25 (26.9)
No	40 (75.5)	28 (70.0)	68 (73.1)
CPIS, *n* (%)			
≤ 6	6 (11.3)	2 (5.0)	8 (8.6)
7	4 (7.5)	8 (20.0)	12 (12.9)
8	10 (18.9)	5 (12.5)	15 (16.1)
> 8	33 (62.3)	25 (62.5)	58 (62.4)
Duration of prior hospitalization^b^ (days)			
< 5, *n* (%)	8 (15.1)	5 (12.5)	13 (14.0)
≥ 5, *n* (%)	43 (81.1)	35 (87.5)	78 (83.9)
Missing, *n* (%)	2 (3.8)	0	2 (2.2)
Mean (SD)	12.0 (8.4)	11.0 (7.3)	11.5 (7.9)
Median (range)	10.0 (2, 44)	9.0 (1, 41)	9.0 (1, 44)
Duration of prior mechanical ventilation^b^ (days)			
< 5, *n* (%)	21 (39.6)	16 (40.0)	37 (39.8)
≥ 5, *n* (%)	31 (58.5)	23 (57.5)	54 (58.1)
Missing, *n* (%)	1 (1.9)	1 (2.5)	2 (2.2)
Mean (SD)	7.3 (5.6)	8.4 (6.8)	7.7 (6.1)
Median (range)	6.4 (0.2, 26.0)	7.3 (0.8, 25.7)	6.6 (0.2, 26.0)
PaO_2_/FiO_2_, mm Hg, *n* (%)			
≤ 240	41 (77.4)	23 (57.5)	64 (68.8)
> 240	12 (22.6)	16 (40.0)	28 (30.1)
Missing	0	1 (2.5)	1 (1.1)
Bacteremia (any pathogen), *n* (%)			
Yes	2 (3.8)	5 (12.5)	7 (7.5)
No	51 (96.2)	35 (87.5)	86 (92.5)
Number of baseline LRT pathogens, *n* (%)			
None confirmed	4 (7.5)	8 (20.0)	12 (12.9)
Monomicrobial	32 (60.4)	21 (52.5)	53 (57.0)
Polymicrobial	17 (32.1)	11 (27.5)	28 (30.1)
Vasopressor use, *n* (%)			
Yes	23 (43.4)	15 (37.5)	38 (40.9)
No	30 (56.6)	25 (62.5)	55 (59.1)

Participants included represent the ITT population

*APACHE* Acute Physiology and Chronic Health Evaluation, *CPIS* Clinical Pulmonary Infection Score, *C/T* Ceftolozane/tazobactam, *ICU* Intensive care unit, *LRT* Lower respiratory tract, *ITT* Intention-to-treat, *MEM* Meropenem, *NP* Nosocomial pneumonia,* PaO*_2_/*FiO*_2_ Arterial oxygen partial pressure to fractional inspired oxygen, *SOFA* Sequential Organ Failure Assessment, *vHABP* Ventilated hospital-acquired bacterial pneumonia, *VABP* Ventilator-associated bacterial pneumonia

^a^Defined as adjunctive empirical therapy with amikacin, which was protocol permitted for up to 72 h at study sites where ≥ 15% of *P. aeruginosa* isolates were resistant to meropenem according to the site’s most recent antibiogram

^b^Assessed prior to randomization

Gram-negative LRT pathogens were identified in 80 ITT participants who were failing prior antibacterial therapy, with a higher proportion of positive cultures in the ceftolozane/tazobactam arm compared with the meropenem arm (92.5% [49/53] vs 77.5% [31/40]). The most common gram-negative isolates identified across both treatment arms were *Klebsiella pneumonia*, *P. aeruginosa*, *Escherichia coli*, and *A. baumannii*. Among participants who were failing prior antibacterial therapy, ESBL + Enterobacterales, *E. coli*, and *P. aeruginosa* were present in > 10% more participants in the ceftolozane/tazobactam arm, otherwise baseline LRT pathogens were comparably distributed in the ITT population (Table 3).

**Table 3:** Baseline LRT pathogens: ASPECT-NP participants who were failing prior antibacterial therapy

ITT population (primary efficacy population)^a^			
LRT pathogen, *n* (%)^b^	C/T (*N* = 53)	MEM (*N* = 40)	Total (*N* = 93)
Any LRT pathogen	49 (92.5)	32 (80.0)	81 (87.1)
Gram-negative	49 (92.5)	31 (77.5)	80 (86.0)
*Pseudomonas aeruginosa*	13 (24.5)	5 (12.5)	18 (19.4)
Enterobacterales	37 (69.8)	21 (52.5)	58 (62.4)
ESBL + Enterobacterales	16 (30.2)	8 (20.0)	24 (25.8)
*Klebsiella pneumoniae*	19 (35.8)	14 (35.0)	33 (35.5)
ESBL + *Klebsiella pneumoniae*	14 (26.4)	8 (20.0)	22 (23.7)
*Escherichia coli*	11 (20.8)	3 (7.5)	14 (15.1)
ESBL + *Escherichia coli*	3 (5.7)	0	3 (3.2)
*Proteus mirabilis*	5 (9.4)	1 (2.5)	6 (6.5)
ESBL + *Proteus mirabilis*	3 (5.7)	1 (2.5)	4 (4.3)
*Serratia marcescens*	3 (5.7)	2 (5.0)	5 (5.4)
*Klebsiella aerogenes*	3 (5.7)	1 (2.5)	4 (4.3)
*Acinetobacter baumannii*	11 (20.8)	10 (25.0)	21 (22.6)
*Stenotrophomonas maltophilia*	0	2 (5.0)	2 (2.2)
No LRT pathogen identified	4 (7.5)	8 (20.0)	12 (12.9)

mITT population (secondary efficacy population)^c^			
LRT pathogen, *n* (%)^b^	C/T (*N* = 39)	MEM (*N* = 24)	Total (*N* = 63)
Any LRT pathogen	39 (100)	24 (100)	63 (100)
Gram-negative	39 (100)	24 (100)	63 (100)
*Pseudomonas aeruginosa*	11 (28.2)	4 (16.7)	15 (23.8)
Enterobacterales	30 (76.9)	17 (70.8)	47 (74.6)
ESBL + Enterobacterales	10 (25.6)	6 (25.0)	16 (25.4)
*Enterobacter cloacae*	2 (5.1)	2 (8.3)	4 (6.3)
*Escherichia coli*	10 (25.6)	2 (8.3)	12 (19.0)
ESBL + *Escherichia coli*	2 (5.1)	0	2 (3.2)
*Klebsiella (Enterobacter) aerogenes*	2 (5.1)	1 (4.2)	3 (4.8)
*Klebsiella pneumoniae*	12 (30.8)	11 (45.8)	23 (36.5)
ESBL + *Klebsiella pneumoniae*	7 (17.9)	6 (25.0)	13 (20.6)
*Proteus mirabilis*	5 (12.8)	1 (4.2)	6 (9.5)
ESBL + *Proteus mirabilis*	3 (7.7)	1 (4.2)	4 (6.3)
*Serratia marcescens*	3 (7.7)	2 (8.3)	5 (7.9)
*Acinetobacter baumannii*	4 (10.3)	3 (12.5)	7 (11.1)
*Stenotrophomonas maltophilia*	0	2 (8.3)	2 (3.2)
No LRT pathogen identified	0	0	0

*C/T* Ceftolozane/tazobactam, *ESBL* Extended-spectrum β-lactamase, *ITT* Intention-to-treat, *LRT* Lower respiratory tract, *MEM* Meropenem, *mITT* Microbiologic intention-to-treat, *n* Number of study participants with the specific pathogen, *N* Number of study participants in the specific treatment arm and analysis population with ≥ 1 baseline LRT

^a^Study participants were eligible for inclusion into the ITT population regardless of whether they had a baseline pathogen, the type of pathogen, and pathogen susceptibility

^b^Incidence ≥ 5%

^c^Study participants were eligible for inclusion into the mITT population only if baseline LRT cultures yielded ≥ 1 gram-negative or streptococcal respiratory pathogen that was susceptible to ≥ 1 study drug

Among those who were failing prior antibacterial therapy, a greater proportion of gram-negative baseline pathogens isolated from participants randomized to the ceftolozane/tazobactam arm were nonsusceptible to the antibacterial therapy administered within 72 h of starting study treatment compared with the meropenem arm (Additional file 1: Table S2). A total of 35 participants had ≥ 1 baseline pathogen nonsusceptible to randomized study drug (23/48 [47.9%] ceftolozane/tazobactam-treated and 12/31 [38.7%] meropenem-treated participants). Additional file 1: Fig. S1 shows the distribution of minimum inhibitory concentration (MIC) values for Enterobacterales and *P. aeruginosa* baseline LRT isolates from participants who were failing prior antibacterial therapy and received meropenem. In total, 23/31 (74.2%) isolates had a meropenem MIC of ≤ 0.25 μg/mL, including 21/25 (84.0%) Enterobacterales and 2/6 (33.3%) *P. aeruginosa* isolates. Multidrug-resistant *P. aeruginosa* was identified in 7/48 (14.6%) and 2/31(6.5%) of participants in the ceftolozane/tazobactam and meropenem arms, respectively, with extensively multidrug-resistant *P. aeruginosa* found in 5/48 (10.4%) ceftolozane/tazobactam-treated and 1/31 (3.2%) meropenem-treated participants.

In the subgroup of participants who were failing prior antibacterial therapy for vHABP/VABP, all ITT participants received ≥ 1 dose of study drug and comprised the safety population. The median (range) treatment duration was 7.7 (0.7, 13.8) days in the ceftolozane/tazobactam arm and 7.7 (0.0, 13.7) days in the meropenem arm, with study treatment received for ≤ 5 days in 11/53 (20.8%) and 11/40 (27.5%) participants, respectively.

### Treatment outcomes

**Fig. 2:**
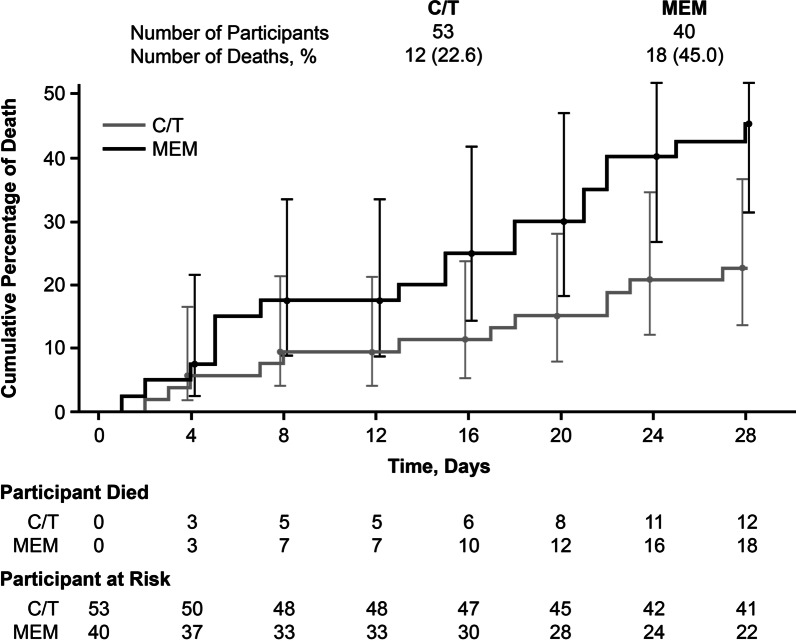
Time to death in participants with ventilator hospital-acquired/ventilator-associated bacterial pneumonia who were failing prior antibacterial therapy (ITT population). *C/T* Ceftolozane/tazobactam, *ITT* Intention-to-treat, *MEM* Meropenem

Table 4 describes rates of mortality, clinical response, and microbiologic response in the subgroup who were failing prior antibacterial therapy. Compared with those who received ceftolozane/tazobactam, mortality was 22.4% higher in participants who received meropenem (18/40 [45.0%] vs 12/53 [22.6%]; 95% CI for the percentage difference: 3.1 to 40.1) in the ITT population. A similar result was observed within the mITT population. The trend toward higher mortality in the meropenem arm was observed beginning on day 2 and continued through day 28 (Fig. 2). In participants who were failing prior antibacterial therapy, clinical response at TOC was 11.6% higher in the ceftolozane/tazobactam arm compared with the meropenem arm; however, the 95% CI for this difference included zero (26/53 [49.1%] vs 15/40 [37.5%]; 95% CI for the percentage difference: − 8.6 to 30.2), with comparable microbiologic response rates at TOC observed between treatment arms in the mITT population (26/39 [66.7%] vs 16/24 [66.7%]; percentage difference [95% CI]: 0.0 [− 22.0 to 23.7]). Mortality rates were similar across treatment arms in those who were not failing prior antibacterial therapy (ceftolozane/tazobactam: 75/309 [24.3%]; meropenem: 74/323 [22.9%]; percentage difference [95% CI]: − 1.4% [− 8.0 to 5.2]). Safety was generally comparable between treatment arms (Additional file 1: Tables S3 and S4). Drug-related AEs (DRAEs) occurred in 4/53 (7.5%) and 1/40 (2.5%) participants in the ceftolozane/tazobactam and meropenem arms, respectively; no serious DRAEs or DRAEs resulting in death occurred in either group.

**Table 4:** Efficacy outcomes in ASPECT-NP participants who were failing prior antibacterial therapy

Endpoint	C/T *n*/*N* (%)	MEM *n*/*N* (%)	% Difference (95% CI)^a^
28-day all-cause mortality (ITT)^b^	12/53 (22.6)	18/40 (45.0)	22.4 (3.1 to 40.1)
28-day all-cause mortality (mITT)^b^	7/39 (17.9)	11/24 (45.8)	27.9 (4.7 to 49.0)
Clinical cure at TOC (ITT)^b^	26/53 (49.1)	15/40 (37.5)	11.6 (− 8.6 to 30.2)
Clinical cure at TOC (CE)^c^	21/33 (63.6)	9/20 (45.0)	18.6 (− 8.2 to 42.5)
Microbiologic eradication at TOC (mITT)^b,d^	26/39 (66.7)	16/24 (66.7)	0.0 (− 22.0 to 23.7)
Microbiologic eradication at TOC (ME)^b,d^	10/17 (58.8)	4/7 (57.1)	1.7 (− 33.7 to 39.3)

*CE* Clinically evaluable, *C/T* Ceftolozane/tazobactam, *ITT* Intention-to-treat, *ME* Microbiologically evaluable, *MEM* Meropenem, *mITT* Microbiological intention-to-treat, *TOC* Test of cure, *n* Number of study participants meeting the criteria for each assessment, *N* Number of study participants in each subgroup of the respective analysis population

^a^Unstratified Newcombe CIs; positive differences are in favor of ceftolozane/tazobactam, negative differences are in favor of meropenem

^b^Participants with missing/indeterminate data were reported as deceased or as failures, depending on the endpoint

^c^Data reported as observed, i.e., participants with missing/indeterminate responses were excluded from analysis

^d^Per-participant microbiologic eradication

### Multivariable analysis

Random forest model runs with a median (interquartile range) out of bag error rate of 33.7% (33.7%–34.8%) ranked the 16 preselected variables from most to least influential toward 28-day ACM. The variables were input into the forward regression model in the same order as the ranking determined using the random forest model analysis. Area under the ROC within forward regression increased from 0.71 to 0.78 with inclusion of the 4 top-ranking variables (i.e., concomitant vasopressor use, baseline age, baseline APACHE II score, and treatment) into the model; addition of other variables resulted in a decrease in ROC. Goodness-of-fit was demonstrated using the Hosmer–Lemeshow test, which yielded a nonsignificant *P* value of 0.8443. Therefore, variable selection indicated that the 4 most influential factors affecting 28-day ACM in the failing prior antibacterial therapy subgroup were concomitant vasopressor use (categorical variable), baseline age (continuous variable), baseline APACHE II score (continuous variable), and treatment (categorical variable).

The magnitude of the relationship of the four variables to mortality was assessed using a backward elimination logistic main effects regression model. Both baseline APACHE II score and age were removed from the model owing to lack of significance. Treatment and vasopressor use remained significant (*P* < 0.05) in the final regression model, indicating a significant relationship with mortality in the failing prior antibacterial therapy subgroup. Area under the ROC curve for the final model was 0.78, indicating successful prediction of mortality was achieved. ORs (95% CIs) for no vasopressor use versus concomitant vasopressor use (adjusting for treatment) and ceftolozane/tazobactam versus meropenem treatment (adjusting for vasopressor use) were 0.12 (0.04 to 0.34) and 0.23 (0.08 to 0.68), respectively (Table 5). The sensitivity analysis confirmed the results observed with the main multivariable analysis, with meropenem treatment and vasopressor use significantly associated with 28-day ACM.

**Table 5:** ORs for risk of 28-day ACM for the final logistic regression model

Participant characteristic	OR for 28-day ACM (95% CI)
No vasopressor use versus concomitant vasopressor use^a^	0.12 (0.04 to 0.34)
Ceftolozane/tazobactam treatment versus meropenem treatment^b^	0.23 (0.08 to 0.68)

OR estimates and CIs associated with the significant factors included into the final logistic regression model, each adjusted for the other factors

*ACM* All-cause mortality, *OR* Odds ratio

^a^Adjusting for treatment

^b^Adjusting for vasopressor use

## Discussion

In the ASPECT-NP trial, ceftolozane/tazobactam was noninferior to meropenem for the treatment of vHABP and VABP for both the primary (28-day ACM) and key secondary endpoints (clinical response at TOC) [24]. The ASPECT-NP study population included participants who were failing antibacterial therapy for vHABP/VABP at the time of enrollment, an important predefined subgroup that may have been at higher risk of mortality because of a refractory response to initial therapy [7, 28]. Delay in initiation of effective antibacterial therapy for the treatment of serious bacterial infections (including nosocomial pneumonia) has consistently been associated with increased mortality [9, 10]. Thus, a higher mortality rate is not unexpected in participants who had received > 48 h of ineffective therapy before receiving study drug than the rest of the study population, who received ≤ 24 h of standard of care antibacterial therapy prior to receiving study therapy. However, increased mortality in this subgroup of participants who were failing prior therapy was only seen in the meropenem treatment arm. Participants in this subgroup who received ceftolozane/tazobactam had lower mortality rates in both the ITT and mITT populations compared with participants who received meropenem. Kaplan–Meier analysis of mortality differences between treatment arms in this subgroup demonstrated divergence between ceftolozane/tazobactam and meropenem treatment, beginning at approximately day 2 and continuing throughout the treatment period, which coincides with the expected timing for failure of antibacterial treatment [32].

Clinical response rates at the TOC visit were higher in the ceftolozane/tazobactam treatment group for both the ITT and CE population; however, the 95% CI for these differences included zero, indicating no statistically significant difference. The reason for a statistically significant difference seen only in the mortality endpoint but not the clinical response rate is uncertain. The relative merits of these 2 endpoints for use in HABP/VABP trials has been a source of continued debate [33]. While clinical response rates may reflect a more disease-specific endpoint than 28-day ACM, it also lacks a standard definition between studies and is based on the subjective assessment of the investigator. These differing attributes may have contributed to the difference in outcomes seen between the 2 endpoints in this study.

Multivariable analysis of this subgroup, which included clinical characteristics, baseline microbiology, and treatment arm, was conducted to determine which factors were independently associated with risk of mortality. Not surprisingly, vasopressor use, a marker of hemodynamic instability and disease severity [34], was the factor most strongly associated with a higher mortality risk independent of treatment arm. However, after controlling for vasopressor use, treatment arm remained strongly associated with mortality risk: treatment with ceftolozane/tazobactam was protective, with an OR of death of 0.23 compared with meropenem treatment. Although this analysis rigorously controlled for confounding factors potentially associated with mortality, these findings should be confirmed with an adequately powered trial. Notably, the ASPECT-NP study evaluated all-cause mortality rather than mortality attributable to pneumonia. As is commonly the case in HABP/VABP studies, ASPECT-NP participants were often critically ill with diseases other than pneumonia, confounding determination of the role of the current pneumonia episode versus participants’ underlying factors in deaths occurring in this study. Although confounding factors should have been largely controlled for by the randomized study design, the possibility remains that the mortality differences between treatment groups were driven by a lower-than-expected rate of non-pneumonia-related mortality in the ceftolozane/tazobactam arm.

Resistance of an infecting pathogen to the chosen treatment is a common cause for failure of antibacterial therapy [8–10]. In this study, we did not have the LRT culture results necessary to determine whether resistance was the reason for antibacterial failure in this subgroup. Our analysis could suggest that antibacterial resistance in the meropenem group might be a contributor to the differential mortality results. However, among those who were failing prior antibacterial therapy, more participants in the ceftolozane/tazobactam arm had ≥ 1 pathogen that were nonsusceptible to study drug compared with the meropenem arm. Moreover, in this subgroup, ESBL-positive Enterobacterales, multidrug-resistant *P. aeruginosa*, and extensively multidrug-resistant *P. aeruginosa* were more prevalent in participants in the ceftolozane/tazobactam arm than in the meropenem arm. Thus, the distribution of resistant pathogens does not explain the higher rate of survival in the ceftolozane/tazobactam treatment arm. Of note, a larger proportion of participants in the meropenem treatment arm had negative LRT cultures at study enrollment. It is possible that persistent signs and symptoms of disease in participants with negative baseline LRT cultures who were failing prior therapy were not caused by bacterial pneumonia that was refractory to antibacterial therapy but were instead related to another disease process in these participants with complex critical illness. However, the treatment-associated mortality difference was also seen in the mITT population, a population with more evidence supporting a diagnosis of pneumonia that was failing therapy, owing to the exclusion of participants with negative baseline LRT cultures.

Another possible explanation for antibacterial treatment failure is underdosing. In the ASPECT-NP study, meropenem was dosed at 1 g every 8 h, per treatment guidelines available at the time of study design [1]. More recently, high-dose meropenem (2 g every 8 h by extended 3-h infusions) has become increasingly recommended in patients with nosocomial pneumonia who are critically ill [29, 35, 36]. This dosing strategy may be particularly beneficial in patients infected with pathogens with MICs at the higher end of the susceptible range and increased β-lactam clearance due to augmented renal clearance [35]. However, in this study, approximately 77% of relevant LRT isolates at baseline had MIC values ≤ 0.25 μg/mL for meropenem suggesting that exposure to high-dose meropenem would likely not have conferred clinical benefit on the majority of participants. In addition, participants in the ASPECT-NP study with augmented renal clearance had similar outcomes to those with normal renal function, suggesting the meropenem dosing was adequate and that alternative meropenem dosing would have had minimal impact on the outcomes within this subgroup [37].

Strengths of these analyses include the underlying strengths of the ASPECT-NP study, which enrolled a population with high disease acuity, representative of a critically ill clinical population for which ceftolozane/tazobactam treatment might be considered [24]. The study included participants who were failing standard of care therapy for HABP/VABP, a common clinical scenario, and yet a subgroup that is often excluded from randomized comparative HABP/VABP trials [24]. The most common prior antibacterial agents administered in this group were reflective of commonly recommended regimens, making our results highly generalizable to clinical practice [1]. Limitations of these analyses include the retrospective design, the small number of participants who were failing prior antibacterial therapy, the lack of information regarding the appropriateness of the initial antibiotic treatment, and the low frequency of some baseline factors, such as bacteremia. Lastly, there is the potential that mortality in both treatment arms within the failing prior antibacterial therapy subgroup was impacted by non-pneumonia-related deaths or that unidentified baseline characteristics were imbalanced between treatment arms, leading to observed differences in outcomes. Although the potential for unidentified imbalances in baseline characteristics leading to the rapid divergence in survival curves observed by day 2 as an alternative explanation for the difference in mortality between treatment arms exists, the robust stratification and randomization processes used in ASPECT-NP make this unlikely. Nevertheless, adequately powered prospective studies are needed to confirm the potential survival advantage conferred by ceftolozane/tazobactam over meropenem in participants with vHABP/VABP who were failing prior antibacterial therapy.

## Conclusions

Multivariable analysis provided further support for the previously demonstrated lower rate of 28-day ACM in the ceftolozane/tazobactam versus the meropenem treatment arm among ASPECT-NP participants who were failing prior antibacterial therapy at study enrollment [24]. When adjusting for other factors that significantly impacted mortality within this population, participants from the failing prior therapy subgroup who received ceftolozane/tazobactam were one-fourth as likely to die versus those receiving meropenem. Based on the findings from this retrospective analysis, ceftolozane/tazobactam may provide a survival advantage over meropenem within this population of patients with treatment-refractory vHABP/VABP, despite the lack of significant differences in clinical cure rate between the treatment arms.

## Supplementary Information

**Additional file 1. Figure S1.** Frequency distribution of meropenem MIC values for **A** Enterobacterales (*N* = 25) and **B** *Pseudomonas aeruginosa* (*N *= 6) isolates obtained from participants with vHABP/VABP who were failing prior antibacterial therapy at study entry and were randomized to the meropenem treatment arm. **Table S1.** Baseline demographics and clinical characteristics in ASPECT-NP participants (ITT population) who were not failing prior antibacterial therapy at study entry. **Table S2.** Nonsusceptibility to failed prior antibacterial therapy among baseline LRT pathogens within 72 h of starting study treatment (ITT population). **Table S3.** AEs in ASPECT-NP participants with ventilated hospital-acquired/ventilator-associated bacterial pneumonia who were failing prior antibacterial therapy. **Table S4.** AEs by System Organ Class and Preferred Term (in ≥ 2 participants) in ASPECT-NP participants with ventilated hospital-acquired/ventilator-associated bacterial pneumonia who were failing prior antibacterial therapy by treatment arm (APaT).

## Data Availability

The data sharing policy, including restrictions, of Merck Sharp & Dohme LLC, a subsidiary of Merck & Co., Inc., Rahway, NJ, USA is available at http://engagezone.msd.com/ds_documentation.php. Requests for access to the clinical study data can be submitted through the Engage Zone site or via email to dataaccess@merck.com.

## Abbreviations

ACM: All-cause mortality
APACHE: Acute Physiology and Chronic Health Evaluation
CE: Clinically evaluable
CFU: Colony-forming unit
CLSI: Current Clinical and Laboratory Standards Institute
CPIS: Clinical Pulmonary Infection Score
DRAE: Drug-related adverse event
EOT: End of therapy
ESBLs: Extended-spectrum β-lactamases
HABP/VABP: Hospital-acquired/ventilator-associated bacterial pneumonia
ITT: Intention-to-treat
IV: Intravenously
LRT: Lower respiratory tract
ME: Microbiologically evaluable
MIC: Minimum inhibitory concentration
mITT: Microbiologic intention-to-treat
OR: Odds ratio
PaO_2_/FiO_2_: Arterial oxygen partial pressure to fractional inspired oxygen
ROC: Receiver operating characteristic
SOFA: Sequential Organ Failure Assessment
TOC: Test of cure
vHABP: Ventilated hospital-acquired bacterial pneumonia
