# Supplementary material for: Outcomes in participants with failure of initial antibacterial therapy for hospital-acquired/ventilator-associated bacterial pneumonia prior to enrollment in the randomized, controlled phase 3 ASPECT-NP trial of ceftolozane/tazobactam versus meropenem
Source: Crit Care. 2022 Dec 1;26:373. doi: 10.1186/s13054-022-04192-w (PMC9714015; doi:10.1186/s13054-022-04192-w)
Supplement: Supplementary file 1 — Additional file 1. Figure S1. Frequency distribution of meropenem MIC values for A Enterobacterales (N = 25) and B Pseudomonas aeruginosa (N = 6) isolates obtained from participants with vHABP/VABP who were failing prior antibacterial therapy at study entry and were randomized to the meropenem treatment arm. Table S1. Baseline demographics and clinical characteristics in ASPECT-NP participants (ITT population) who were not failing prior antibacterial therapy at study entry. Table S2. Nonsusceptibility to failed prior antibacterial therapy among baseline LRT pathogens within 72 h of starting study treatment (ITT population). Table S3. AEs in ASPECT-NP participants with ventilated hospital-acquired/ventilator-associated bacterial pneumonia who were failing prior antibacterial therapy. Table S4. AEs by System Organ Class and Preferred Term (in ≥ 2 participants) in ASPECT-NP participants with ventilated hospital-acquired/ventilator-associated bacterial pneumonia who were failing prior antibacterial therapy by treatment arm (APaT). [file 13054_2022_4192_MOESM1_ESM.docx]

# SUPPLEMENTAL APPENDIX

Figure S1. Frequency distribution of meropenem MIC values for (A) Enterobacterales (*N* = 25) and (B) *Pseudomonas aeruginosa* (*N* = 6) isolates obtained from participants with vHABP/VABP who were failing prior antibacterial therapy at study entry and were randomized to the meropenem treatment arm. Breakpoints indicated reflect 2021 (current) CLSI susceptibility breakpoints for meropenem. CLSI, Current Clinical and Laboratory Standards Institute; MIC, minimum inhibitory concentration; vHABP/VABP, ventilated hospital-acquired/ventilator-associated bacterial pneumonia.

1. Enterobacterales (CLSI susceptibility breakpoint for meropenem: 1 µg/mL)

1. *Pseudomonas aeruginosa* (CLSI susceptibility breakpoint for meropenem: 2 µg/mL)

Table S1. Baseline demographics and clinical characteristics in ASPECT-NP participants (ITT population) who were not failing prior antibacterial therapy at study entry

|  | **C/T**  **(*N* = 309)** | **MEM**  **(*N* = 323)** | **Total**  **(*N* = 632)** |
| --- | --- | --- | --- |
| Primary diagnosis, *n* (%) |  |  |  |
| VABP | 230 (74.4) | 233 (72.1) | 463 (73.3) |
| vHABP | 79 (25.6) | 90 (27.9) | 169 (26.7) |
| Sex, *n* (%) |  |  |  |
| Male | 219 (70.9) | 230 (71.2) | 449 (71.0) |
| Female | 90 (29.1) | 93 (28.8) | 183 (29.0) |
| Age, years |  |  |  |
| <65, *n (%)* | 176 (57.0) | 183 (56.7) | 359 (56.8) |
| ≥65, *n (%)* | 133 (43.0) | 140 (43.3) | 273 (43.2) |
| Mean (SD) | 60.5 (16.3) | 59.4 (17.3) | 60.0 (16.8) |
| Median (range) | 62.0 (18, 98) | 62.0 (18, 90) | 62.0 (18, 98) |
| Weight, kg |  |  |  |
| Median (range) | 80.0 (41.0, 150.1) | 80.0 (40.0, 151.0) | 80.0 (40.0, 151.0) |
| Body-mass index, kg/m^2^ |  |  |  |
| Median (range) | 27.0 (16.4, 49.3) | 26.6 (15.5, 56.0) | 26.7 (15.5, 56.0) |
| Creatinine clearance, mL/min, *n* (%) |  |  |  |
| ≥150 (augmented renal clearance) | 58 (18.8) | 58 (18.0) | 116 (18.4) |
| ≥80 | 192 (62.1) | 210 (65.0) | 402 (63.6) |
| <80 to >50 | 71 (23.0) | 68 (21.1) | 139 (22.0) |
| ≤50 to ≥30 | 30 (9.7) | 23 (7.1) | 53 (8.4) |
| <30 to ≥15 | 15 (4.9) | 19 (5.9) | 34 (5.4) |
| <15 (end-stage renal disease) | 0 | 1 (0.3) | 1 (0.2) |
| Missing | 1 (0.3) | 2 (0.6) | 3 (0.5) |
| APACHE II score |  |  |  |
| ≤14, *n (%)* | 76 (24.6) | 80 (24.8) | 156 (24.7) |
| 15–19, *n (%)* | 131 (42.4) | 136 (42.1) | 267 (42.2) |
| ≥20, *n (%)* | 101 (32.7) | 105 (32.5) | 206 (32.6) |
| Missing, *n (%)* | 1 (0.3) | 2 (0.6) | 3 (0.5) |
| Mean (SD) | 17.4 (5.2) | 17.4 (5.8) | 17.4 (5.5) |
| Median (range) | 17.0 (2, 33) | 17.0 (2, 39) | 17.0 (2, 39) |
| SOFA score, *n (%)* |  |  |  |
| ≤7 | 225 (72.8) | 203 (62.8) | 428 (67.7) |
| >7 | 84 (27.2) | 119 (36.8) | 203 (32.1) |
| Missing | 0 | 1 (0.3) | 1 (0.2) |
| Prior nonstudy gram-negative therapy,^a^ *n* (%) | | | |
| Yes | 265 (85.8) | 283 (87.6) | 548 (86.7) |
| No | 44 (14.2) | 40 (12.4) | 84 (13.3) |
| Adjunctive gram-negative therapy,^b^ *n (%)* | | | |
| Yes | 90 (29.1) | 100 (31.0) | 190 (30.1) |
| No | 218 (70.6) | 218 (67.5) | 436 (69.0) |
| Missing | 1 (0.3) | 5 (1.5) | 6 (0.9) |
| CPIS, *n* (%) |  |  |  |
| ≤6 | 19 (6.1) | 30 (9.3) | 49 (7.8) |
| 7 | 25 (8.1) | 27 (8.4) | 52 (8.2) |
| 8 | 35 (11.3) | 37 (11.5) | 72 (11.4) |
| >8 | 230 (74.4) | 229 (70.9) | 459 (72.6) |
| Duration of prior hospitalization,^c^ days |  |  |  |
| <5, *n (%)* | 72 (23.3) | 76 (23.5) | 148 (23.4) |
| ≥5, *n (%)* | 235 (76.1) | 244 (75.5) | 479 (75.8) |
| Missing, *n (%)* | 2 (0.6) | 3 (0.9) | 5 (0.8) |
| Mean (SD) | 10.8 (24.7) | 9.8 (10.8) | 10.2 (18.9) |
| Median (range) | 7.0 (1, 418) | 7.0 (1, 116) | 7.0 (1, 418) |
| Duration of prior mechanical ventilation,^c^ days | | | |
| <5, *n (%)* | 157 (50.8) | 168 (52.0) | 325 (51.4) |
| ≥5, *n (%)* | 151 (48.9) | 153 (47.4) | 304 (48.1) |
| Missing, *n (%)* | 1 (0.3) | 2 (0.6) | 3 (0.5) |
| Mean (SD) | 10.8 (55.9) | 6.8 (9.2) | 8.8 (39.7) |
| Median (range) | 4.9 (0.02, 767.4) | 4.8 (0.04, 107.5) | 4.9 (0.02, 767.4) |
| PaO_2_/FiO_2_, mm Hg, *n* (%) |  |  |  |
| ≤240 | 225 (72.8) | 242 (74.9) | 467 (73.9) |
| >240 | 81 (26.2) | 80 (24.8) | 161 (25.5) |
| Missing | 3 (1.0) | 1 (0.3) | 4 (0.6) |
| Bacteremia (any pathogen), *n* (%) |  |  |  |
| Yes | 62 (20.1) | 36 (11.1) | 98 (15.5) |
| No | 247 (79.9) | 287 (88.9) | 534 (84.5) |
| Number of baseline LRT pathogens, *n* (%) | | | |
| None confirmed | 39 (12.6) | 48 (14.9) | 87 (13.8) |
| Monomicrobial | 145 (46.9) | 160 (49.5) | 305 (48.3) |
| Polymicrobial | 125 (40.5) | 115 (35.6) | 240 (38.0) |

APACHE, Acute Physiology and Chronic Health Evaluation; CPIS, Clinical Pulmonary Infection Score; C/T, ceftolozane/tazobactam; ITT, intention-to-treat; LRT, lower respiratory tract; MEM, meropenem; NP, nosocomial pneumonia; PaO_2_/FiO_2_, arterial oxygen partial pressure to fractional inspired oxygen; SOFA; Sequential Organ Failure Assessment; VABP, ventilator-associated bacterial pneumonia; vHABP, ventilated hospital-acquired bacterial pneumonia.

^a^Antibacterial therapy active against gram negative pathogens received in the 72 hours prior to first dose of study drug.

^b^Defined as adjunctive empirical therapy with amikacin, which was protocol permitted for up to 72 hours at study sites where ≥15% of *P. aeruginosa* isolates were resistant to meropenem according to the site’s most recent antibiogram.

^c^Assessed prior to randomization.

Table S2. Nonsusceptibility to failed prior antibacterial therapy among baseline LRT pathogens within 72 hours of starting study treatment (ITT population)

|  | **C/T** | **MEM** |
| --- | --- | --- |
|  | **(*N* = 53)** | **(*N* = 40)** |
|  | ***n/N1* (%)** | ***n/N1* (%)** |
| Gram-negative | 48/71 (67.6) | 24/45 (53.3) |
| *Pseudomonas aeruginosa* | 8/13 (61.5) | 2/5 (40.0) |
| AmpC-overexpressing *Pseudomonas aeruginosa* | 1/2 (50.0) | 0 |
| Enterobacterales | 30/46 (65.2) | 10/24 (41.7) |
| ESBL+ Enterobacterales | 19/21 (90.5) | 7/9 (77.8) |
| *Citrobacter koseri* | 0/1 (0.0) | 0 |
| *Enterobacter cloacae* | 2/2 (100.0) | 0/2 (0.0) |
| *Escherichia coli* | 3/11 (27.3) | 0/2 (0.0) |
| ESBL+ *Escherichia coli* | 3/3 (100.0) | 0 |
| *Klebsiella aerogenes* | 3/3 (100.0) | 1/1 (100.0) |
| *Klebsiella oxytoca* | 1/1 (100.0) | 0/1 (0.0) |
| *Klebsiella pneumoniae* | 15/19 (78.9) | 7/14 (50.0) |
| ESBL+ *Klebsiella pneumoniae* | 13/14 (92.9) | 6/8 (75.0) |
| *Klebsiella sp* | 0 | 1/1 (100.0) |
| *Pantoea agglomerans* | 1/1 (100.0) | 0 |
| *Proteus mirabilis* | 3/5 (60.0) | 1/1 (100.0) |
| ESBL+ *Proteus mirabilis* | 2/3 (66.7) | 1/1 (100.0) |
| *Serratia marcescens* | 2/3 (66.7) | 0/2 (0.0) |
| ESBL+ *Serratia marcescens* | 1/1 (100.0) | 0 |
| Other | 10/12 (83.3) | 12/16 (75.0) |
| *Achromobacter xylosoxidans* | 0 | 1/1 (100.0) |
| *Acinetobacter baumannii* | 10/11 (90.9) | 9/10 (90.0) |
| *Acinetobacter lwoffii* | 0 | 0/1 (0.0) |
| *Delftia acidovorans* | 0 | 0/1 (0.0) |
| *Elizabethkingia meningosepticum* | 0 | 0/1 (0.0) |
| *Haemophilus influenzae* | 0/1 (0.0) | 0 |
| *Stenotrophomonas maltophilia* | 0 | 2/2 (100.0) |

C/T, ceftolozane/tazobactam; ESBL, extended-spectrum β-lactamase; ITT, intention-to-treat; LRT, lower respiratory tract; MEM, meropenem; *n*, the number of pathogens within a specific category; *N*, the number of participants in the population; *N1*, the number of pathogens with baseline susceptibility data available.

Table S3. AEs in ASPECT-NP participants with ventilated hospital-acquired/ventilator-associated bacterial pneumonia who were failing prior antibacterial therapy

| **AE category, *n* (%)** | **C/T**  **(*N* = 53)** | **MEM**  **(*N* = 40)** | **Total**  **(*N* = 93)** |
| --- | --- | --- | --- |
| Participants with ≥1 TEAE | 48 (90.6) | 32 (80.0) | 80 (86.0) |
| Participants with ≥1 TEAE by maximum severity^a^ | | | |
| Mild | 11 (20.8) | 3 (7.5) | 14 (15.1) |
| Moderate | 16 (30.2) | 7 (17.5) | 23 (24.7) |
| Severe | 21 (39.6) | 22 (55.0) | 43 (46.2) |
| Participants with ≥1 DRAE^b^ | 4 (7.5) | 1 (2.5) | 5 (5.4) |
| Participants with ≥1 serious TEAE | 20 (37.7) | 21 (52.5) | 41 (44.1) |
| Participants with ≥1 serious DRAE^b^ | 0 | 0 | 0 |
| Participants with ≥1 TEAE leading to study drug discontinuation | 5 (9.4) | 8 (20.0) | 13 (14.0) |
| Participants with ≥1 DRAE leading to study drug discontinuation^b^ | 0 | 1 (2.5) | 1 (1.1) |
| Participants with ≥1 TEAE resulting in death | 15 (28.3) | 18 (45.0) | 33 (35.5) |
| Participants with ≥1 DRAE resulting in death^b^ | 0 | 0 | 0 |

AEs were coded using MedDRA version 17.0. For each category, participants were counted only once, even if they experienced multiple events in the category

AE, adverse event; C/T, ceftolozane/tazobactam; DRAE, drug-related adverse event; MedDRA, Medical Dictionary for Regulatory Activities; MEM, meropenem; *n*, number of participants in specific category; *N*, number of participants in safety population; TEAE, treatment-emergent adverse event (defined as any AE starting on/after the first study drug administration).

^a^If participants had multiple events, they were counted only once with the maximum (highest) severity rating. The orders of severity from worst case to best case were severe, moderate, and mild.

^b^If a participant had multiple events and ≥1 was deemed related to study drug, then that participant was included in the count.

Table S4. AEs by System Organ Class and Preferred Term (in ≥2 participants) in ASPECT-NP participants with ventilated hospital-acquired/ventilator-associated bacterial pneumonia who were failing prior antibacterial therapy by treatment arm (APaT)^a^

| **AE category, *n* (%)** | **C/T**  **(*N* = 53)** | **MEM**  **(*N* = 40)** | **Total**  **(*N* = 93)** |
| --- | --- | --- | --- |
| Blood and lymphatic system disorders | | | |
| Anemia | 3 (5.7) | 3 (7.5) | 6 (6.5) |
| Thrombocytopenia | 0 | 4 (10.0) | 4 (4.3) |
| Cardiac disorders | | | |
| Bradycardia | 0 | 2 (5.0) | 2 (2.2) |
| Cardiac arrest | 1 (1.9) | 2 (5.0) | 3 (3.2) |
| Cardiac failure acute | 3 (5.7) | 1 (2.5) | 4 (4.3) |
| Gastrointestinal disorders | | | |
| Diarrhea | 3 (5.7) | 0 | 3 (3.2) |
| Upper gastrointestinal hemorrhage | 2 (3.8) | 1 (2.5) | 3 (3.2) |
| Hepatobiliary disorders | | | |
| Cholecystitis | 0 | 2 (5.0) | 2 (2.2) |
| Infections and infestations | | | |
| Bacteremia | 2 (3.8) | 1 (2.5) | 3 (3.2) |
| Conjunctivitis | 1 (1.9) | 2 (5.0) | 3 (3.2) |
| Genitourinary tract infection | 2 (3.8) | 0 | 2 (2.2) |
| Pneumonia | 2 (3.8) | 1 (2.5) | 3 (3.2) |
| Septic shock | 2 (3.8) | 3 (7.5) | 5 (5.4) |
| Sinusitis | 1 (1.9) | 2 (5.0) | 3 (3.2) |
| Urinary tract infection | 3 (5.7) | 2 (5.0) | 5 (5.4) |
| Investigations | | | |
| Alanine aminotransferase increased | 5 (9.4) | 1 (2.5) | 6 (6.5) |
| Aspartate aminotransferase increased | 6 (11.3) | 1 (2.5) | 7 (7.5) |
| Liver function test abnormal | 2 (3.8) | 0 | 2 (2.2) |
| Metabolism and nutrition disorders | | | |
| Hyperglycemia | 2 (3.8) | 0 | 2 (2.2) |
| Hypernatremia | 3 (5.7) | 1 (2.5) | 4 (4.3) |
| Hypoalbuminemia | 2 (3.8) | 0 | 2 (2.2) |
| Malnutrition | 0 | 2 (5.0) | 2 (2.2) |
| Musculoskeletal and connective tissue disorders | | | |
| Musculoskeletal pain | 0 | 2 (5.0) | 2 (2.2) |
| Nervous system disorders | | | |
| Brain midline shift | 2 (3.8) | 0 | 2 (2.2) |
| Quadriparesis | 3 (5.7) | 0 | 3 (3.2) |
| Psychiatric disorders | | | |
| Agitation | 2 (3.8) | 0 | 2 (2.2) |
| Delirium | 2 (3.8) | 0 | 2 (2.2) |
| Renal and urinary disorders | | | |
| Polyuria | 5 (9.4) | 1 (2.5) | 6 (6.5) |
| Renal failure acute | 3 (5.7) | 2 (5.0) | 5 (5.4) |
| Respiratory, thoracic and mediastinal disorders | | | |
| Aspiration | 2 (3.8) | 1 (2.5) | 3 (3.2) |
| Hydrothorax | 4 (7.5) | 2 (5.0) | 6 (6.5) |
| Pneumothorax | 3 (5.7) | 1 (2.5) | 4 (4.3) |
| Respiratory failure | 2 (3.8) | 1 (2.5) | 3 (3.2) |
| Skin and subcutaneous tissue disorders | | | |
| Decubitus ulcer | 5 (9.4) | 2 (5.0) | 7 (7.5) |
| Surgical and medical procedures | | | |
| Tracheostomy | 2 (3.8) | 0 | 2 (2.2) |
| Vascular disorders | | | |
| Deep vein thrombosis | 0 | 2 (5.0) | 2 (2.2) |
| Hypertensive crisis | 0 | 2 (5.0) | 2 (2.2) |
| Hypotension | 4 (7.5) | 1 (2.5) | 5 (5.4) |

AEs were coded using MedDRA version 17.0. For each category, participants were counted only once, even if they experienced multiple events in the category

AE, adverse event; APaT, all participants as treated; C/T, ceftolozane/tazobactam; DRAE, drug-related adverse event; MedDRA, Medical Dictionary for Regulatory Activities; MEM, meropenem; TEAE, treatment-emergent adverse event (defined as any AE starting on/after the first study drug administration).

^a^Limited to AEs present in ≥2 participants in either treatment arm.
